# Good’s Syndrome With Pure Red Cell Aplasia and Subclinical Myasthenia Gravis: A Case Report and Review of Literature

**DOI:** 10.7759/cureus.59654

**Published:** 2024-05-04

**Authors:** Wakana Hashiro, Akihiro Miyashita, Yuka Kawaji-Kanayama, Haruya Okamoto, Takahiro Fujino, Taku Tsukamoto, Shinsuke Mizutani, Yuji Shimura, Junya Kuroda

**Affiliations:** 1 Division of Hematology and Oncology, Department of Medicine, Kyoto Prefectural University of Medicine, Kyoto, JPN

**Keywords:** cyclosporine, pure red cell aplasia, myasthenia gravis, immunoglobulin disorder, hypogammaglobulinemia, thymoma, good’s syndrome

## Abstract

Good's syndrome is a pathologic condition characterized by thymoma and immunoglobulin disorder. Here, we report a rare case of a patient with Good’s syndrome with simultaneous pure red cell aplasia (PRCA) and subclinical myasthenia gravis with detectable serum anti-acetylcholine receptor antibody (AChR Ab). While thymectomy did not result in the improvement of any paraneoplastic syndromes, cyclosporine A (CsA) treatment successfully improved PRCA; however, hypoglobulinemia was not recovered, and anti-AchR Ab did not disappear by CsA treatment in our case. A review of the literature on simultaneous Good’s syndrome with PRCA also suggested the efficacy of CsA on PRCA but not hypoglobulinemia, suggesting the distinct underlying mechanisms between these two paraneoplastic symptoms with thymoma. Future research is needed to understand the mechanism underlying this rare pathologic condition and to generate appropriate treatment.

## Introduction

Patients with thymoma are frequently afflicted by various paraneoplastic manifestations, including myasthenia gravis (MG), pure red cell aplasia (PRCA), Lambert-Eaton syndrome, bullous pemphigoid, systemic lupus erythematosus, and ulcerative colitis. MG has been the most commonly observed with thymoma among those while PRCA has been observed in about 3-10% of cases [1,2]. In addition to these, Good’s syndrome is one of the adult-onset pathological conditions complicated with thymoma, which is characterized by concurrent immunoglobulin disorder representing hypogammaglobulinemia or agammaglobulinemia and recurrent infection due to immunodeficiency [2-6]. While Goods’ syndrome may be identified in approximately 6-11% of patients with thymoma [6], simultaneous presentation with Good's syndrome, PRCA, and MG is extremely rare. In addition, the differential diagnosis from various diseases for anemia is frequently required for the diagnosis of Good's syndrome. Therefore, the clinical manifestation and optimal therapeutic management for this condition have not been established. We herein report a clinical feature and treatment course of a patient with Good's syndrome complicated by PRCA and MG.

## Case presentation

**Figure 1:**
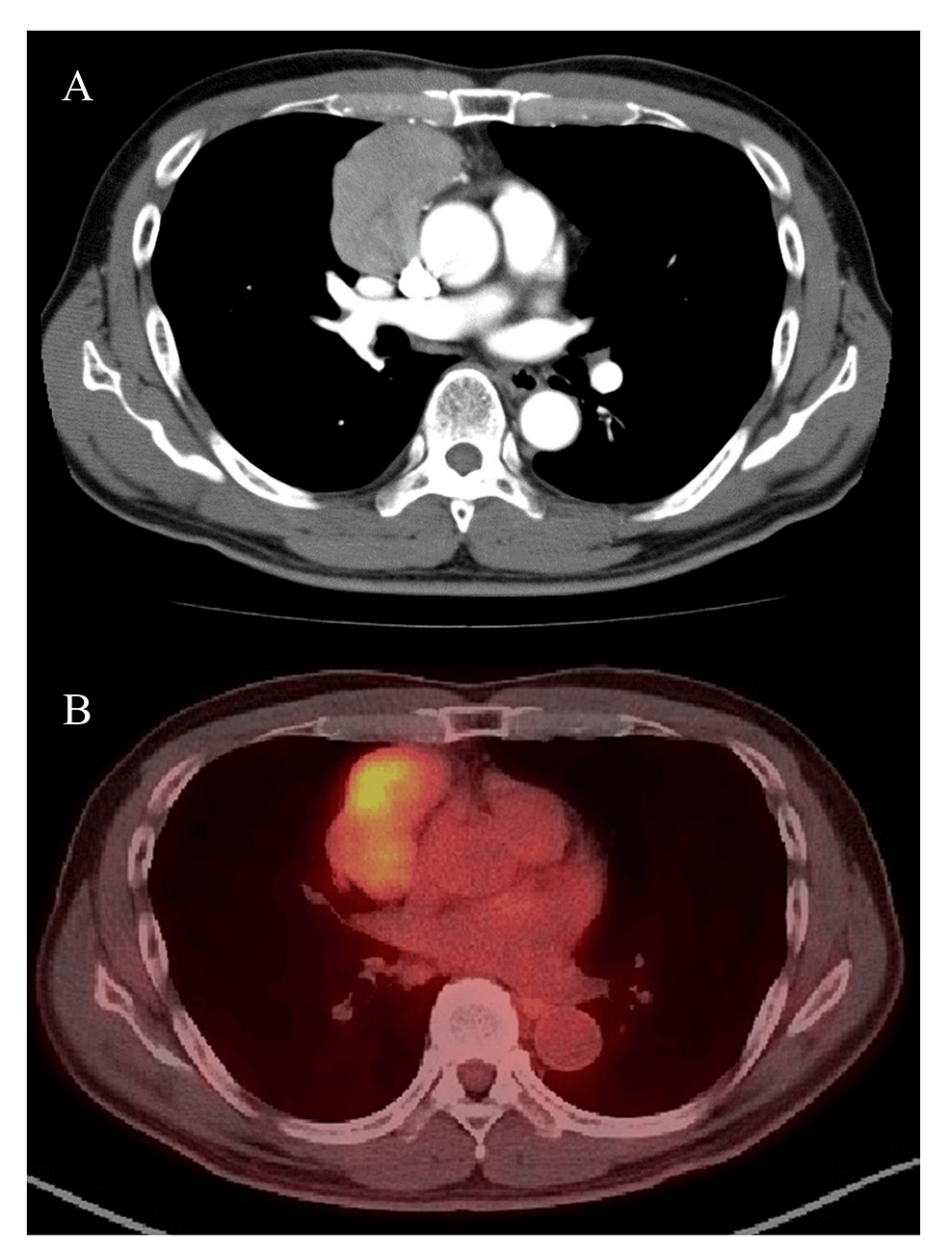
Radiologic findings at diagnosis A. Contrast-enhanced computed tomography shows a poorly contrasted anterior mediastinal tumor. B. ^18^F-fluorodeoxyglucose (FDG) positron emission tomography showed an FDG-avid anterior mediastinal tumor.

A 71-year-old male patient was referred to our institute for the diagnosis and treatment of an anterior mediastinal tumor, which was 5.9 cm in diameter and was positive for ^18^F-fluorodeoxyglucose-positron emission tomography with a maximum standardized uptake value of 5.0 (Figure 1). He was complaining of shortness of breath during exertion without muscle weakness. His medical history included otitis media at 19 years old and an open bone fracture of the right lower extremity at 63 years old while he had no immunological abnormality.

**Figure 2:**
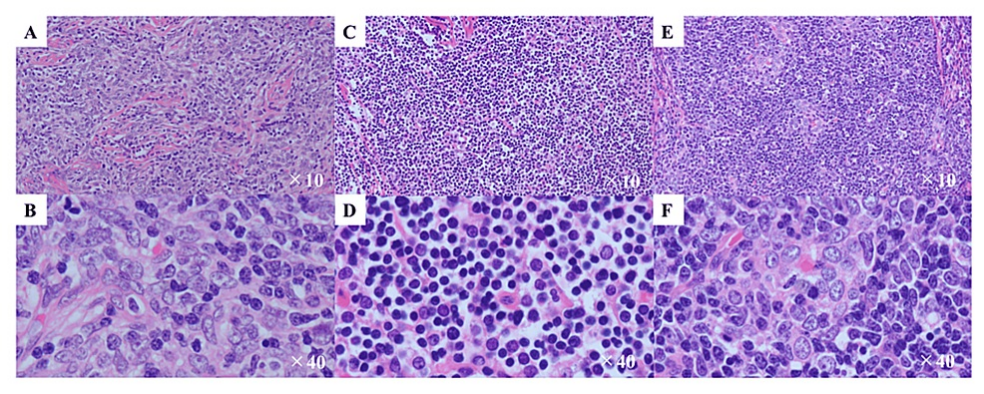
Histopathological findings of hematoxylin-eosin-stained biopsied specimens of the anterior mediastinal tumor A and B. A and B showed a part of thymoma that was composed of a few lymphocytes and proliferating spindle cells, which was consistent with the diagnosis of Type A thymoma based on the WHO classification [7] (A: x10 view and B: x40 view). C and D. C and D show the different parts of the thymoma, mainly composed of lymphocytes, consistent with Type B (C: x10 view and D: x40 view). E and F. The rest of the lesions of the thymoma showed the feature of Type AB thymoma where Type A and Type B were mixed (E: x10 view and F: x40 view). Accordingly, the patient was diagnosed as having Type AB thymoma.

The histopathological assessment of biopsied specimens of thymoma disclosed the mixed presence of type A, type B, and type AB thymomas according to the World Health Organization classification [7], reaching the diagnosis of type AB thymoma (Figure 2).

**Figure 3:**
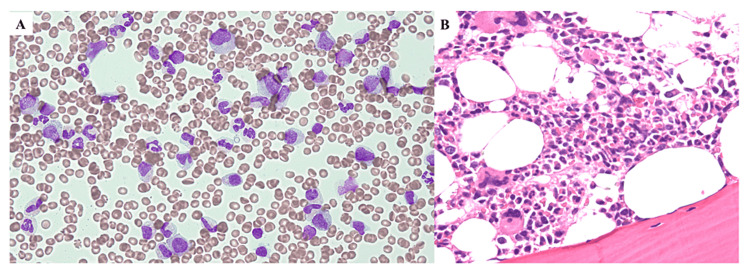
Cytologic and histologic findings of bone marrow A. Bone marrow smear stained by May-Giemsa staining. B. Hematoxylin-eosin-stained bone marrow biopsied specimen.

At that time, the blood examination disclosed normocytic anemia with the hemoglobin level decreased to 7.6 g/dL (normal range: 13.7-16.8g/dL) with a mean corpuscular volume of 84 fL (normal range: 83.6-98.2 fL) and a reduced number of reticulocytes of 4.5 x 10^9^/L (normal range: 25.0-75.0 x 10^9^/L). White blood cell count was within the normal range (5.1 x 10^9^/L), including 0.786 x 10^9^/L of CD4-positive T lymphocytes (normal range: 0.7-1.3 x 10^9^/L). CD4- and CD8-positive T lymphocyte ratio was 1.1 (normal range: 0.6-2.9). The number of B lymphocytes was 0.190 x 10^9^/L in peripheral blood (normal range: 0.1-0.6 x 10^9^/L). The platelet count was normal. The serological test showed an increase in serum ferritin to 938 μg/dL (normal range: 20-250 μg/dL) and decreased immunoglobulin (Ig) G to 674 mg/dL (normal range: 861-1747 mg/dL), IgM to 26 mg/dL (normal range: 50-299 mg/dL), and IgA to 74 mg/dL (normal range: 93-393 mg/dL). The serum protein fraction showed a pattern of slight hypoglobulinemia without evidence of monoclonal protein. In addition, the anti-acetylcholine receptor antibody was increased to 4.8 nmol/L (normal range:≦0.2 nmol/L). Other serological tests showed no abnormality, including lactate dehydrogenase, bilirubin, haptoglobin, and C-reactive protein. The bone marrow aspiration findings showed normocellular marrow with a nucleated cell count of 101.0 ×10^9^/L (normal range: 100-250 ×10^9^/L); however, erythroblasts were absent. In addition, the ratios of myeloblast and lymphocytes were 1.6% and 14.0%, respectively, while no dysplasia was observed, consistent with the diagnosis of PRCA (Figure 3). Accordingly, the patient was finally diagnosed as having Good’s syndrome with concomitant PRCA and subclinical MG.

**Figure 4:**
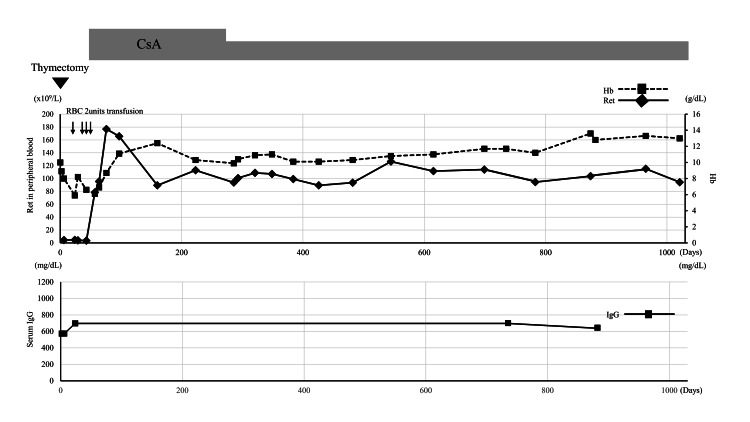
Post-thymectomy clinical course CsA: cyclosporine A, Hb; hemoglobin; IgG; immunoglobulin G, RBC: red blood cell concentrate. Ret: reticulocyte

Because the thymectomy did not induce recovery from PRCA for a month, the patient was subjected to treatment with 3 mg/kg/day of cyclosporin A (CsA), which caused the sustained recovery of reticulocytes and the subsequent recovery of anemia within two months (Figure 4). In contrast, 1100 days after the operation, the hypogammaglobulinemia was not improved, and the anti-acetylcholine receptor antibody remained positive. Fortunately, the patient has not experienced infectious complications with the prophylactic use of acyclovir and sulfamethoxazole-trimethoprim but no supplement of intravenous immunoglobulin.

## Discussion

Paraneoplastic syndrome has been reported to occur in 30-40% of patients with thymoma. MG is the most common, occurring in approximately 20-30% while PRCA and immunoglobulin disorder have been reported to co-occur with thymoma in 1.6-5% and 6-11% of patients, respectively. In contrast, it has been reported that approximately half of patients with PRCA possess thymoma [3,6]. However, the co-occurrence of more than two paraneoplastic syndromes with thymoma is infrequent. When we sought to investigate the clinical manifestations, recent treatment procedures, and treatment response for patients with Good’s syndrome with simultaneous PRCA reported during these two decades, we found only 13 reported cases, including ours (Table 1) [8-18]. Moreover, except for our patient, we found only three patients with Good’s syndrome with simultaneous PRCA and clinical or subclinical MG [9,15,16].

**Table 1:** Reported cases with simultaneous presentation of Good’s syndrome and PRCA since 2000 *: thymectomy plus irradiation, **: thymectomy plus systemic chemotherapy M: male, F: female, CsA: cyclosporin A, CS: corticosteroids, IgG: immunoglobulin G, Hb: hemoglobin, AIHA: autoimmune hemolytic anemia, MG: myasthenia gravis, GS: Good’s syndrome, PRCA: pure red cell aplasia, Ref.: reference number. NA: data not available

Case	Age/Sex	Thymoma type	Data at diagnosis		Other paraneoplastic symptoms	Treatment		Improvement by thymectomy		Improvement by immunosuppressive		First author (Ref)
			IgG (mg/dL)	Hb (g/dL)		Thymectomy	for GS and PRCA (postoperative)	GS	PRCA	GS	PRCA	
1	56/M	AB	320	4.7 mmol/L	-	+	-	-	+	NA	NA	van der Marel J [8]
2	46/F	AB	675	5.0	Subclinical MG	+	CS	-	+	NA	NA	Lin CS [9]
3	55/M	B1	479	5.6	-	+**	-	-	-	NA	NA	Briones J [10]
4	55/F	AB	354	4.9	-	+	CsA and CS	-	-	NA	+	Shiraishi J [11]
5	57/F	AB	241	5.3	-	+	-	-	+	NA	NA	Taniguchi T [12]
6	79/F	AB	Approx. 500	3.7	-	+	CsA	-	-	+	+	Kuribayashi K [13]
7	NA/F	Invasive AB	197	3.9	-	+*	-	-	+	NA	NA	Chen J [14]
8	90/M	B1	291	3.7	MG	-	Rituximab	NA	NA	NA	+	Antar AI [15]
9	50/M	AB	343	6.3	MG	+	CsA	-	-	-	+	Okui M [16]
10	50-55/F	AB	NA.	4.5	-	+	CsA and CS	-	-	NA	+	Yen CC [17]
11	50-55/F	AB	NA.	5.8	AIHA	+	-	-	-	NA	NA	Yen CC [17]
12	56/F	B2	405	5.2	Multiorgan autoimmunity	+**	CsA	-	-	-	+	Nakagawa Y [18]
13	71/M	AB	674	7.6	Subclinical MG	+	CsA	-	-	-	+	Present case

Reviewing the 13 patients with Good’s syndrome with PRCA provokes us to discuss several issues. First, with available data, PRCA was improved by thymectomy in 4 of 12 patients. Although this illustrates the limited therapeutic impact of thymectomy on PRCA, this finding at the same time shows thymectomy as the first treatment approach in Good’s syndrome patients with PRCA and the need to watch and wait without immunosuppressive therapy after thymectomy to see if spontaneous improvement of PRCA occurs. The second is about alternative treatment. The immunosuppressive therapy with CsA appeared highly effective for PRCA accompanied by Good’s syndrome while corticosteroid was also used in three patients. It was intriguing that the anti-CD20 antibody rituximab effectively improved PRCA with Good’s syndrome and MG in one previous case [15]. The therapeutic efficacy of rituximab on PRCA has also been reported with that complicated with B cell lymphoproliferative disorders [19]. However, considering its B cell depletion activity, it remains questionable whether rituximab treatment is genuinely appropriate for patients with Good’s syndrome, which not infrequently induces fetal opportunistic infections due to immunosuppressive status. Thus, rituximab treatment may be better considered as the salvage therapy for patients resistant to or intolerant to CsA. Fortunately, our case was not complicated by infection, but as noted above, Good’s syndrome is associated with immunodeficiency, and preventing infectious complications is also critical. Thus, especially for those patients treated with immunosuppressive therapy, prophylactic options must be considered, including annual supplemental gamma globulin medication, antibiotics, and antivirals. Third, in contrast to cases complicated by PRCA alone, all patients who also had MG were found to be unresponsive to thymectomy and CsA treatment. In addition, although the existing data are somewhat limited, additional therapy by anti-cholinesterase drugs may be needed for symptomatic MG with Good’s syndrome. Indeed, the anti-acetylcholine receptor antibody remains positive after CsA treatment in our patient. In addition, CsA was ineffective in controlling MG in a previous patient with Good’s syndrome with PRCA and MG [16]. Finally, we found that the histologic subtypes in most patients were classified as low-risk, i.e., AB to B1, but high-risk subtypes may also exist. Nevertheless, more information is needed to establish the optimal therapeutic strategy for this rare pathologic condition.

The inconsistent response to CsA between hypoglobulinemia, PRCA, and MG may be related to the mechanisms underlying each pathologic condition. In Good's syndrome, the impairment of both cell-mediated immunity by T lymphocyte dysregulation and humoral immunity by B lymphocyte dysregulation co-exist. In fact, regarding impaired cell-mediated immunity, patients with Good's syndrome occasionally present abnormal CD4+/CD8+ T lymphocyte ratio and CD4 T lymphopenia, which were normal in our case [3,4]. Although the pathogenesis, including genetic factors, remains fully unveiled, it has been reported that the specific clonal expansion of Vβ8-positive CD8-positive T cells and the decrease of interleukin-4-producing Th2 type T cells may cause B lymphopenia [20]. Considering the efficacy of CsA treatment and thymectomy for PRCA, PRCA seems more dependent on T cell dysregulation in this pathologic condition; however, T cell modulation by CsA may not lead to the subsequent improvement of hypoglobulinemia and MG.

## Conclusions

In conclusion, we report a rare case of Good’s syndrome with PRCA and subclinical MG. After reviewing recent case reports for a similar condition within two decades, we determined the therapeutic efficacy of thymectomy and CsA treatment for PRCA but not hypoglobulinemia and MG. Future research is needed to understand the mechanism underlying this rare pathologic condition and to generate appropriate treatment.
